# State of the art on the physical mapping of the Y-chromosome in the Bovidae and comparison with other species — A review

**DOI:** 10.5713/ab.21.0480

**Published:** 2022-03-02

**Authors:** Cristina Rossetti, Viviana Genualdo, Domenico Incarnato, Filomena Mottola, Angela Perucatti, Alfredo Pauciullo

**Affiliations:** 1 Laboratory of Animal Cytogenetics and Genomics, National Research Council (CNR), ISPAAM, 80056 Portici (Napoli), Italy; 2 Department of Environmental, Biological and Pharmaceutical Sciences and Technologies, University of Campania “Luigi Vanvitelli”, 81100 Caserta, Italy; 3 Department of Agricultural, Forest and Food Sciences, University of Turin, 10095 Grugliasco (TO), Italy

**Keywords:** Bovidae, Fluorescence *In situ* Hybridization, Genes, Y-chromosome

## Abstract

The next generation sequencing has significantly contributed to clarify the genome structure of many species of zootechnical interest. However, to date, some portions of the genome, especially those linked to a heterogametic nature such as the Y chromosome, are difficult to assemble and many gaps are still present. It is well known that the fluorescence *in situ* hybridization (FISH) is an excellent tool for identifying genes unequivocably mapped on chromosomes. Therefore, FISH can contribute to the localization of unplaced genome sequences, as well as to correct assembly errors generated by comparative bioinformatics. To this end, it is necessary to have starting points; therefore, in this study, we reviewed the physically mapped genes on the Y chromosome of cattle, buffalo, sheep, goats, pigs, horses and alpacas. A total of 208 *loci* were currently mapped by FISH. 89 were located in the male-specific region of the Y chromosome (MSY) and 119 were identified in the pseudoautosomal region (PAR). The *loci* reported in MSY and PAR were respectively: 18 and 25 in *Bos taurus*, 5 and 7 in *Bubalus bubalis*, 5 and 24 in *Ovis aries*, 5 and 19 in *Capra hircus*, 10 and 16 in *Sus scrofa*, 46 and 18 in *Equus caballus*. While in Vicugna pacos only 10 *loci* are reported in the PAR region. The correct knowledge and assembly of all genome sequences, including those of genes mapped on the Y chromosome, will help to elucidate their biological processes, as well as to discover and exploit potentially epistasis effects useful for selection breeding programs.

## INTRODUCTION

In the last years, the genome of different farm animals, including pig [1], cow [2], buffalo [3], sheep [4], goat [5], horse [6], and camel [7], has been sequenced. Furthermore, there are species like the alpaca that have progressed slowly in the assembly because of their difficult karyotype [8,9]. The data obtained from these studies have significantly contributed to the understanding of domestication [10], to the selection of better breeds [11,12] and to the interaction between genetic traits and the environment [13,14].

In the studies of mammalian genome and in particular of livestock species, the sex chromosomes (X and Y) have considerable importance. Especially the Y-chromosomes (Chr Y), which have undergone substantial evolutionary changes and losing about 95% of the ancestral genesis [15]. The Chr Y is the smallest chromosome and consists of 2% to 3% of the haploid genome and, according to the species, may contain between 70 and 200 genes. It is involved in the segregation of the sex chromosomes in male meiosis. Therefore, it plays a key role for evolutionary studies, speciation, male infertility and/or subfertility due to its unique features, such as long non-recombining regions (NRYs), abundance of repetitive sequences, and holandric inheritance pattern [16]. This chromosome is generally separated into two distinct domains: the pseudoautosomal region (PAR) and the non-pseudoautosomal region, also known as the male-specific region of the Y chromosome (MSY). The PAR is a characteristic DNA region distinct from the autosomes that exhibits sequence homology between sex chromosomes [17].

The MSY region is not subject to pairing during meiosis. Therefore, it has been considered a NRY, although abundant recombination has been reported in humans [18,19]. Furthermore, the MSY contains gene families encoding multi-copy proteins associated with male fertility [15,20].

To date, Chr Y sequencing has been completed and characterized only in some species, including the human [18], the chimpanzee, the rhesus macaque [21], the mouse [22], the pig [23] and the horse [24]. Conversely, data are not available for other species.The molecular cytogenetic techniques, like the fluorescence *in situ* hybridization (FISH), offer a powerful tool in support of the bioinformatics pipelines following next generation sequencing projects. In fact, genomes assemblies are prone to errors, as they have been detected for instance in cattle, goats and sheep genomes by FISH [25,26]. Therefore, the use of the cytogenetic approaches are still fundamental for a correct assembly process. In fact, the FISH by bacterial artificial chromosome (BAC) clones allows assigning sequence contings to specific chromosomes and allows establishing the correct physical order of each DNA fragment, as demonstrated for both animal and vegetable sequencing processes [27,28].

Since the knowledge of the genomes elevates to a different meaning if associated with chromosomes, both for the accuracy of the information and for the evaluation of all related biological aspects [29]; in the present study, we describe the current state of physical gene mapping by FISH in the main species of zootechnical interest (cattle, buffalo, sheep, goat, pig, horse, and alpaca). The choice to focus the attention on this point lies in the current gap of genome assembly data on Chr Y and in the fundamental importance of cytogenetics in the study and interpretation of the genome [30,27,31], as already understood in 1920 by Hans Winkler when he coined the term “genome”.

## BOVIDS Y-CHROMOSOME AND PSEUDOAUTOSOMAL REGION

The Bovidae, in particular we considered *Bos taurus* (BTA), *Bubalus bubalis* (BBU), *Ovis aries* (OAR) and *Capra hircus* (CHI), play a role of fundamental importance for the livestock sector from an economic point of view. For this reason, these species have been investigated deeply from a genetic point of view, in particular for their fertility and consequently their sex chromosomes [32–35].

Although generally small, the Y-chromosome has different size and shape in the bovids. Indeed, in BTA the Chr Y is a small submetacentric, in BBU is a small acrocentric, while in both OAR and CHI is a very small metacentric [36,37]. However, in some breeds of bovids, the Chr Y is fused with an autosome, as in the case of the male of *Gazella granti* [38]. Moreover, the Chr Y shows few bands because it is almost completely heterochromatic. Besides, the pseudoautosomal boundary (PAB) separates the PAR with similar sequences between X and Y-chromosomes, from the MSY of reduced homology and specific to individual sex chromosomes [39]. In bovids, the genes present in the PAR of the Chr Y are the same in content and sequence as in the X chromosome [15].

### Bos taurus

**Figure 1:**
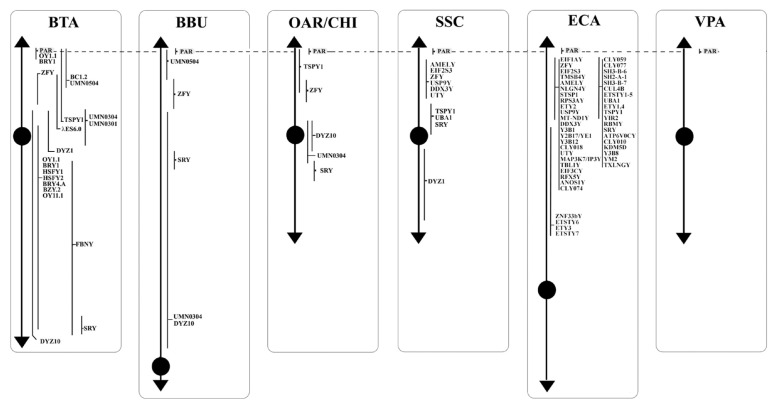
Schematic representation of MSY *loci* along the Y chromosomes of BTA, BBU, OAR, CHI, SSC, ECA, and VPA. MSY, male-specific region of the Y chromosome; BTA, *Bos taurus*; BBU, *Bubalus bubalis*; OAR, *Ovis aries*; CHI, *Capra hircus*; SSC, *Sus scrofa*; ECA, *Equus caballus*; VPA, Vicugna pacos.

The bovine genome was among the first genomes to be partially sequenced, after the sequencing of the human one. The latest cattle genome assembly release (ARS-UCD1.2) of the whole genome, contains 29 pairs of the autosomes, the X chromosome, and unplaced sequences. Information on the Chr Y is not available yet. Through the years, only few studies focused on the physical mapping of molecular markers by FISH [40–48]. These studies allowed confirming the presence and the precise location of eighteen genes on the MSY, as showed in Table 1 and Figure 1.

**Figure 2:**
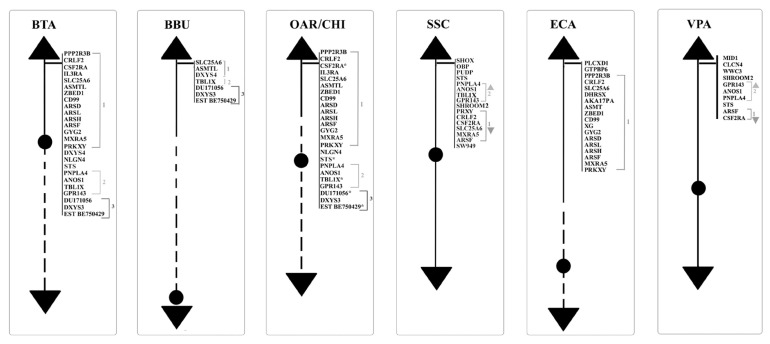
Schematic representation of PAR *loci* along the Y chromosomes of BTA, BBU, OAR, CHI, SSC, ECA, and VPA. PAR, pseudoautosomal region; BTA, *Bos taurus*; BBU, *Bubalus bubalis*; OAR, *Ovis aries*; CHI, *Capra hircus*; SSC, *Sus scrofa*; ECA, *Equus caballus*; VPA, Vicugna pacos.

The PAR region is located at the telomere of the short arm of the Chr Y and contains twenty five genes physically mapped [34,49–51,45,39,52,53], as showed in Table 2 and Figure 2.

### Bubalus bubalis

The buffalo genome has been recently sequenced with a contiguity surpassing both human and goat genomes [3]. The assembly UOA_WB_1 containis 24 pairs of the autosomes, the X chromosome and unplaced sequences. Also for the buffalo, no indications have been reported on Chr Y and, so far, only the studies by FISH allowed correctly placing five genes on the MSY (Table 1, Figure 1) [40,41,43–45, 47,48].

The buffalo PAR is located on the telomere of q-arm and contains seven genes already mapped (Table 2, Figure 2) [45, 39,52,53].

### Ovis aries/Capra hircus

The assembly of OAR and CHI genomes are Oar_rambouillet_v1.0. and Goat CVASU_BBG_1.0, respectively. The presence and precise location of five genes on the MSY were confirmed by FISH (Table 1, Figure 1) [40,41,43–45,47,48].

The PAR is located on the telomere of p-arm and it contains twenty four and nineteen genes physical mapped respectively for OAR and CHI (Table 2, Figure 2) [45,39,52,53].

## PIG Y-CHROMOSOME AND PSEUDOAUTOSOMAL REGION

The domestic pig, *Sus scrofa* (SSC), plays a key role in the meat industry, but it acquired great importance also as biomedical animal model for many human diseases. An updated version of its genome sequencing has been recently published as Sscrofa11.1. Despite the new version, the annotation is fully available for the autosomes and the X chromosome, while little data is available for the Chr Y. Thanks to the cytogenetic investigation by BAC clones and FISH [54–56], it was possible to obtain information about the Y morphology and the evolution of sex chromosome genes. The Chr Y is the smallest of the pig chromosomes, metacentric and constituted of about 50 Mb in length as assessed by flow cytometry [57,58]. Chr Y short arm (Yp) is characterized by the presence of the most male-specific single-copy genes (physical mapped). Furthermore, given the highly repetitive nature of the long arm, to date, only a single copy sequence (*DYZ1*) is mapped on this chromosome portion (Table 1, Figure 1).

Regarding the information of the porcine PAR region, of sixteen *loci* only one is located in the terminal short arms of the sex chromosomes. Currently, the *loci* already known are only those cytogenetically mapped [55]. Moreover, the PAB lies next the shroom family member 2 (*SHROOM2*) or most proximal gene (Figure 2).

## HORSE Y-CHROMOSOME AND PSEUDOAUTOSOMAL REGION

The horse, *Equus caballus* (ECA), is an economically and culturally important domestic species. The equine genome is approximately 2.68 Gb long and there are currently ~1,150 *loci* mapped by FISH with an average of one marker per ~2.5 Mb of the genome [59]. The ECA Y-chromosome is a small submetacentric, and forty-six genes have been mapped, as shown in Table 1 and Figure 1 [60–62,24]. The ECA genome is derived from a female, so the Chr Y has been poorly characterized.

Subsequently, through the sequencing of cDNA libraries, the gene content and the complete map of the euchromatic region were defined [24]. The map covers both the PAR and MSY regions [60,63]. A total of 129 markers, 110 sequence-tagged site (*STS*) and 19 genes, were found in the PAR. This region includes the PAB, which is located between *PRKXY* and *EIF1AY* in the Y chromosome [64]. Studies conducted on ECA have shown the presence of duplicate genes both in MSY and PAR [63,65]. So far, this condition has been observed only in horses and, having no other information, further studies would be necessary to confirm or exclude these duplications also in other equids/perrisodactyls or mammals. To date, the current map contains approximately ~400 BAC clones [66]. In Paria’s study of the 2009, through the use of the direct cDNA selection method, 29 genes and expressed sequence tag (ESTs) were identified and 23 out of them were known to be specific to the horse Y chromosome. To date, a total of 37 genes/transcripts from the horse MSY region were identified and showed that 20 genes are X-degenerate with known orthologs in other Eutherian species. The remaining 17 genes were acquired or novel and identified so far only in the horse or donkey Y chromosomes [62]. The PAR region is located at the telomere of the short p-arm of the X chromosome and at the telomere of the long q-arm of the Y chromosome and contains 18 physical mapping genes (Table 2, Figure 2) [17].

## ALPACA Y-CHROMOSOME AND PSEUDOAUTOSOMAL REGION

The alpaca, Vicugna pacos (VPA), is a species of Camelids originally from South America. The quality of its meat [67, 68], the opportunity to exploit it as a source of milk [69] and, especially, its fiber make this species of fundamental importance for the economy of many countries [70,71].

The reference genome for this species is the Vic.Pac 3.1, which has been annotated for about 90%, and identifies ~76% of the genome to chromosomes [72,73]. The cytogenetic map currently known for the alpaca consists of 281 genes representative of all chromosomes [72]. So far, no genes have been identified by cytogenetic mapping on the Y-chromosome, except for ten *loci* on the PAR Yq-ter region (Table 2; Figure 2) because the CHORI-246 BAC library derives from a female alpaca [74]. The Alpaca Y-chromosome, according to the most recent physical mapping (Jevit et al [75]) is a small submetacentric, the smallest among of Old-World camels [76], and the PAR region is located in the long arm.

## DISCUSSION

In this work, we report on the physical Y-gene mapping by FISH in the main farm animals species. Considering the existing gap in the genome assembly for the Chr Y, the present review is of great importance as one of the first indications to consider for a correct assembly. Moreover, the markers mapped on the Chr Y may offer useful indications for selection and have pratical implication in breeding programs for the biological function they may carry out. In fact, genes located on the Y chromosome are essential for spermatogenesis and male fertility, as demonstrated, for example, in Holstein bulls by association studies between Y-linked gene copy number variations (*PRAMEY*, *HSFY*, and *ZNF*) and fertility traits [77,78].

Although Y chromosome has never been a direct target for selection, fertility is always a significant factor in determining livestock productivity. In the majority of breeding programs for the farm animals, including those treated in the present study, the female-to-male sex ratio is significantly higher than one for the combination of the intensive artificial selection and the use of artificial insemination technology with high breeding value males. This condition greatly reduces the number of blood lineages and increases the consanguinity, as well as, the inbreeding depression on productive and reproductive traits, including fertility. Therefore, addressing this gap of knowledge will also increase the potential use of Y Chr markers for breeding purposes.

### Comparisons of genes mapped on Y-chromosome by FISH among farm animals

To date, using the FISH method, it has been possible to map physically 208 *loci* belonging to the Y-chromosome of the species reviewed in the present study. In particular, 89 *loci* mapped in the non-pseudoautosomal region (Table 1) and 119 in the PAR region (Table 2). Regarding the comparison between PAR regions there is an interesting observation to do. This region is in the distal part of the Y-chromosome for all the species studied, but in BTA/OAR/CHI/SSC it is at the telomere of the p-arm, while in ECA/BBU it was reported at the telomere of the q-arm (Figure 2). In addition, very recently, the same position has been confirmed also in VPA [75]. In particular, in this species, the Y-chromosome is very small and does not show distinct cytogenetic characteristic, so that it is difficult to identify the location of the centromere. Only with the use of the FISH technique by PAR BACs, it has been demonstrated that the alpaca Chr Y is submetacentric with a very small and short p-arm and the PAR located at the telomere in the long q-arm [75].

Seventy-five out of 119 *loci* mapped in the PAR region and reviewed in this study were reported in bovids, whereas the other 44 were mapped in the other species. More in details, seven *loci* were mapped exclusively in the bovids (*ASMTL*, *IL3RA*, *NLGN4*, *DU171056*, *DXYS3*, *DXYS4*, *EST BE750429*) with the *ASMTL* and *DXYS3* identified in all the four species (BTA, BBU, OAR, and CHI). *DXYS4* physically located only in BTA and BBU, *IL3RA* and *NLGN4X* present in all except BBU, *DU171056* and *EST BE750429* absents in CHI. Three *loci* were mapped exclusively in VPA (*CLCN4*, *MID1*, *WWC3*), whereas four were identified only in SSC (*OBP*, *PUDP*, *SW949*, *SHOX*). One locus, *SHROOM2*, was mapped in SSC and VPA, and six were exclusively identified in ECA (*AKAP17A*, *ASMT*, *DHRSX*, *GTPBP6*, *PLCXD1*, *XG*) (Table 2). PAR genes have maintained a high level of synteny and conservation between different species. Indeed, their comparison would lead to divide them into three sub-regions. The first consists of *PPP2R3B*, *CRLF2*, *CSF2RA*, *IL3RA*, *SLC25A6*, *ASMTL*, *ZBED1*, *CD99*, *ARSD*, *ARSL*, *ARSH*, *ARSF*, *GYG2*, *MXRA5*, *PRKXY*, *DXYS4*, and *NLGN4* (sub-region 1), the second *STS*, *PNPLA4*, *ANOS1*, *TBL1X* and *GPR143* (sub-region 2), the third presents only in bovids, with *DU171056*, *DXYS3*, *EST BE750429*.

Sub-region 1 is present in all the species we reviewed and the genes mapped in this area mostly maintained the same order. In fact, the sequences mapped by FISH in bovids and horses maintained synteny, with the exception of *GYG2* in ECA, which is located between *CD99* and *ARSD* and not between *ARSF* and *MXRA5* as identified in bovids (Figure 2). Furthermore, in bovids, BTA and OAR-CHI showed the same genes mapped with the exception of *CSF2RA* present only in OAR, while in BBU, it is possible to find only three genes mapped out of the genes belonging to this subregion. In pig and alpaca, the order of this sub-region is reversed compared to other species, although only some of the sequences in this area have been mapped for both species (Figure 2). Furthermore, within the same sub-region 1 the order of *MXRA5* and *ARSF* for pig, *ARSF* and *CFS2RA* for alpaca do not retain synteny compared to other species.

A similar matter is evident also for the sub-region 2, present in almost all the species of interest except for the ECA. In BTA and OAR-CHI the mapped sequences are the same. Among these, only *TBL1X* was not mapped to CHI and is, instead, the only gene present in BBU. For both pigs and alpacas this area appears in order before sub-region 1 (Figure 2). Furthermore, the *STS* gene in VPA mapped to the end of this area and not to the beginning as it can be found in BTA, OAR and SSC. Regarding the absence of this sub-region in the ECA, Janečka et al [24] reported a transposition of some PAR genes in the MSY region, such as *TBL1Y*, *ANOS1Y*, *STSP1* (Figure 1). This transposition would facilitate the recombination between PAR and MSY. This situation would justify the deletions in the MSY region that can be found in horses with sexual development disorders [24].

About the genes of this subregion, *TBL1X* is implicated in the testosterone concentration, spermatogenesis, and sperm motility; it was used as marker and it resulted important for the predicition of reproductive performance [79].

The sub-region 3, as mentioned above, regards only bovids and it conserved in BTA, BBU, OAR, and CHI a perfect synteny.

**Figure 3:**
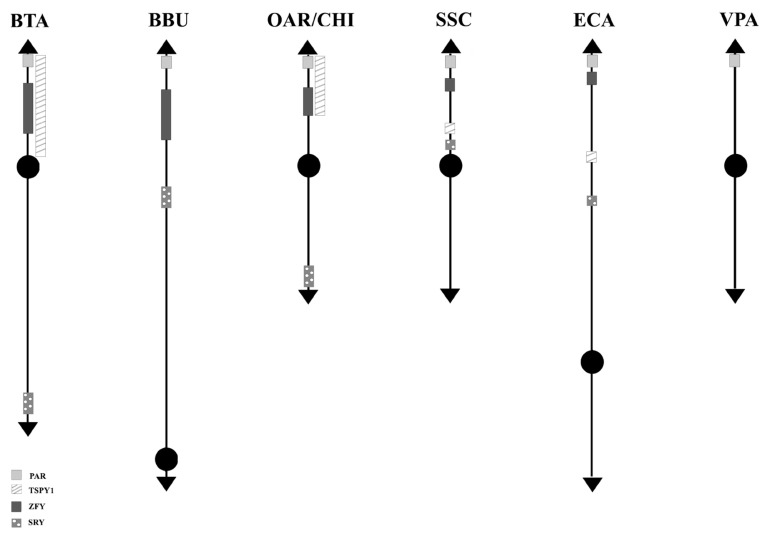
Schematic representation of *SRY-ZFY-TSPY1* loci along the Y-chromosomes. No information has been found in VPA so far. VPA, Vicugna pacos.

Concerning the 89 *loci* FISH mapped in the non pseudoautosomal region of the Chr Y (MSY), ECA represents the species reporting most of the *loci* currently mapped on Chr Y. Alone, it shows 46 positioned *loci* compared to BTA that has 18 mapped *loci*. About the other species, 10 *loci* were reported for SSC and 5 for BBU, OAR, and CHI. No genes were physically mapped to the alpaca Y-chromosome (Figure 1). Moreover, in this region, only the *loci SRY*, *ZFY* and *TSPY1* were identified in most of the species investigated, that is BTA, OAR/CHI, SSC, and ECA; while BBU conserved only *SRY* and *ZFY* (Figure 3). The last two *loci* maintained a good synteny among the species, including in BBU where *ZFY* has been found in the telomeric region. In fact, according to Di Meo et al [45], the BBU Chr Y underwent a pericentric inversion compared to same chromosome in BTA. A similar situation has been described also for *UMN0504* that is located near the PAR region only in these two species [45]. Regarding the locus *TSPY1*, it preserved synteny in the distal area of the short arm [48,42] in BTA, OAR and CHI. Conversely in SSC, this gene has been always reported near the centromeric region of the short arm [54]; and in ECA it has been reported in the euchromatic region of Y q-arm [60].

*SRY*, *ZFY*, and *TSPY1* have been considered very important and used as markers for sexual screening as they are involved in spermatogenesis and sexual differentiation [80–82].

*DYZ1*, a male-specific repeat DNA sequence, has been mapped only in BTA and SSC Y [54]. In particular, concerning BTA, the gene maps in the Yp13-q12 region with a higher concentration in the centromeric region [40], as confirmed by Habermann et al [46].

The *AMELY*, *EIF2S3*, *USP9Y*, *DDX3Y*, *UBA1*, and *UTY* genes were mapped only in SSC and ECA.). In SSC, *AMELY* is the first gene after the PAR region. However, according to Quilter et al [54], the orientation of the *loci* changes in ECA as a result of two rearrangements, so that the genes have the following order: *EIF2S3*, *AMELY*, *USP9Y*, *DDX3Y*, *UTY*, and *UBA1* (Figure 1) [60,62]. Among them, *AMELY* is very important in the selection breeding programs because it represents a marker for the sex diagnosis of abnormalities involving the Y chromosome [83].

Instead *EIF2S3*, *DDX3Y*, *UTY* of this region together with *SHROMM2* and *SRY* of MSY region were investigated for the level of their expression in the amniotic fluid [84]. This information can be very important in early sex determination by ensuring a targeted breeding program in species of economic interest.

*UMN0304* and *DYZ10* were mapped only in bovids. The former *locus* in BTA and OAR/CHI maps to the proximal pericentromeric regions of the p- and q-arms, whereas in BBU covers almost the entire Y chromosome except for the R-positive telomeric band. The latter *locus* maps in BTA and BBU as a painting, and in OAR/CHI to the proximal pericentromeric region of the p- and q-arms [45]. As regards *DYZ10* and its different mapping, it must be considered that the data in literature are outdated in times former than the advent of new molecular technologies that allowed genomic libraries to be screened selectively. It would be interesting to localize again this gene, as well as many others, with new technologies like the PacBio sequencing [85] to be associated with classical methods such as FISH or Fiber-FISH. Concering the latter method, the Fiber-FISH is a technique that allows a significantly higher mapping resolution due to the direct visualization of chromatin fibers released from interphase nuclei that are extremely less condensed than the metaphase chromosomes observed by FISH. The Fiber-FISH is often used for determining size-gapping problems, locating delections, resolving chromatin breakpoints linked to diseases, estimating gene copy number variations, orientations, genes length, etc. [86,87].

Of the remaining genes, twelve were mapped only in cattle, and 37 in horses, as reported in Figure 1. The great interest in this species can be traced back to its ancient origins. The horse is in fact one of the first domesticated species that is integrated in the human working life as well as in his leisure activities [88]. Over the years, the interest in this species has increased because it is used in many rehabilitative activities or because it is used as a reference for the study of many pathologies, also considering the strong synteny between the chromosomes of this species with those of humans [89]. In addition, the fertility of stallions plays an essential role in the equine breeding industry (especially for racehorses), and although fertility is of primary importance for all species of zootechnical interest, this information is often limited also in this species.

From these results, the FISH technique turns out to be still a powerful tool contributing to the reduction of gaps currently present in sequencing processes.

The sequencing processes, in fact, in particular related to the Y-chromosome for livestock species, are still very complex and, in many respects, still little known due to the difficulty of assembling heterogametic genomes and the presence of highly repetitive ampliconic regions [15]. In addition, the sequencing of heterogametic genomes requires greater depth than in the homogametic genomes, resulting in an increase in costs [90]. One of the methods of sequencing sex chromosomes is based on the alignment of the heterogametic genome reads with the homogametic ones of the same species. In this way, those regions that are little reproduced on the homogametic chromosomes, are highlighted and are specific of the heterogametic chromosome [91].

As regards the PAR region, this Y-chromosome part is evolving [92,93], but, so far, only a few reports of PAR comparisons between species other than humans and mice have been undertaken [65,39].

Implementing comparative studies on animal genomes by FISH physical mapping of markers along the chromosomes, it would allow a better understanding of the evolutionary process in the different species, including the discovery of complex rearrangements [45] and the filling up of the current gaps in animal genomes.

Furthermore, the knowledge of a correct Y-genes assembly along the chromosome will give the opportunity to investigate more intensively their epistasis effects [94] for instance in the regulation of autosomal gene expression or in the control of the individual fitness. In fact, as demonstrated in humans [95] and Drosophila [96] the Y-chromosome carries multiple genes that differentially affect the expression of hundreds of X-linked and autosomal genes with a functional impact on microtubule stability, metabolism and spermatogenesis [97]. Such epistatic genetic effect might be considered a consequence of the autosomal nature of both sex chromosomes [98], so that prior to becoming sex chromosomes have been involved in autosome-autosome interactions. Thus, although the Y chromosome is a specialized part of the genome, it can play a key role on autosomal gene expression and individual fitness by interacting with the rest of the genome [99].

## CONCLUSION

In the recent years, the genome of many species has been completed, including farm animals of economic interest. However, portions, mostly concerning the Y chromosome, are still unknown or not correctly assembled. The difficulty lies in the absence of reference points that can validate sequence data generated by Next-Generation Sequencing (NGS). In this respect, a real contribution to reduce the gap of knowledge and possible assembly errors on Chr Y can come from the use of the FISH combined with its derived techniques like the Fiber-FISH. The Fiber-FISH would allow to walk on all chromosome starting from specific markers in order to cover any gap, establish the correct order of very near genes or identify the right location of old and new repeated sequences.

Furthermore, the physical map by FISH could be of support also in the study of the cytological characteristics of the chromatin that controls the gene expression and regulation when combined with immunoassay techniques.

The data reported in this mini-review may be a starting point for further studies and future applications in animal science.

## Figures and Tables

**Table 1:** Cytogenetic chromosome localisation of physically mapped MSY *loci* on the Y-chromosome of BTA, BBU, OAR, CHI, SSC, ECA, and VPA and their references

Locus			Physical localization							References
	
Symbol	Name^*^	Protein type	BTA	BBU	OAR	CHI	SSC	ECA	VPA	
*AMELY*	amelogenin, Y-linked	biomineral tissue development	-	-	-	-	Yp12-p13	Yq14-q15	-	SSC: Quilter et al [54] ECA: Raudsepp et al [60]
*ANOS1Y* ECA: *KAL1*	anosmin 1	peptidase inhibitor activity	-	-	-	-	-	Yq14-q15	-	ECA: Janeka et al [24]
*ATP6V0CY*	ATPase, H + transporting, lysosomal 16kDa, V0 subunit c	membrane transporter	-	-	-	-	-	Yq14-q15	-	ECA: Paria et al [62]
*BC1.2*	DNA segment	-	Yp13-p12	-	-	-	-	-	-	BTA: Goldammer et al [43]
*BRY1*	bovine male specific repeat	-	Yp13 + Yp11-qter	-	-	-	-	-	-	BTA: Thomsen et al [41]
*BRY4.A*	DNA segment	-	Yp11-q12 ter	-	-	-	-	-	-	BTA: Thomsen et al [41]
*BZY.2*	DNA segment	-	Yp11-q12 ter	-	-	-	-	-	-	BTA: Thomsen et al [41]
*CLY010*	DNA linear STS	-	-	-	-	-	-	Yq14	-	ECA: Raudsepp et al [60]
*CLY018*	DNA linear STS	-	-	-	-	-	-	Yq14	-	ECA: Raudsepp et al [60]
*CLY059*	DNA linear STS	-	-	-	-	-	-	Yq14	-	ECA: Raudsepp et al [60]
*CLY074*	DNA linear STS	-	-	-	-	-	-	Yq14	-	ECA: Raudsepp et al [60]
*CLY077*	DNA linear STS	-	-	-	-	-	-	Yq14	-	ECA: Raudsepp et al [60]
*CUL4B* ECA: *CUL4BY*	cullin 4B ECA:Y-linked cullin 4B	ubiquitin-protein transferase activity	-	-	-	-	-	Yq14	-	ECA: Paria et al [62]
*DDX3Y (DBY)* SSC: *DBY* ECA: *DDX3Y*	DEAD-box helicase 3 Y-linked	nucleic acid binding	-	-	-	-	Yp12-p13	Yq14	-	SSC: Quilter et al [54] ECA: Raudsepp et al [60]
*DYZ1*	DNA segment (DYZ-1)	-	Yp11–q11	-	-	-	Yq12	-	-	BTA: Habermann et al [46] SSC: Quilter et al [54]
*DYZ10* BTA-BBU-OAR-CHI: *IDVGA50*	Microsatellite	-	Yp12.1-q12.3	Yq11-q1.10	Yp11-q11	Yp11-q11	-	-	-	BTA-BBU-OAR-CHI: Di Meo et al [45]
*EIF1AY*	translation initiation factor 1A Y	translation initiation factor activity	-	-	-	-	-	Yq14	-	ECA: Paria et al [62]
*EIF2S3* SSC-ECA: *EIF2s3Y*	eukaryotic translation initiation factor 2 subunit gamma	translation factor activity, RNA binding	-	-	-	-	Yp12-p13	Yq14	-	SSC: Quilter et al [54] ECA: Paria et al [62]
*EIF3CY*	eukaryotic translation initiation factor, subunit C on Y	formation of cytoplasmic translation initiation complex	-	-	-	-	-	Yq14	-	ECA: Paria et al [62]
*ETSTY1–5*	equus testis-specific transcript Y1–5	-	-	-	-	-	-	Yq14	-	ECA: Paria et al [62]
*ETSTY6*	equus testis-specific transcript Y6	-	-	-	-	-	-	Yq13 (painting)	-	ECA: Paria et al [62]
*ETSTY7*	equus testis-specific transcript Y7	-	-	-	-	-	-	Yq13 (painting)	-	ECA: Paria et al [62]
*ETY1,4*	Equus (transcript) Y1,4	-	-	-	-	-	-	Yq14	-	ECA: Paria et al [62]
*ETY2*	Equus (transcript) Y2	-	-	-	-	-	-	Yq14	-	ECA: Paria et al [62]
*ETY3*	Equus (transcript) Y3	-	-	-	-	-	-	Yq13 (painting)	-	ECA: Paria et al [62]
*FBNY*	DNA fragment	-	Yq12-qter (painting)	-	-	-	-	-	-	BTA: Weikard et al [44]
*HSFY1*	heat shock transcription factor Y-linked 1	transcriptional activators	Yp11-q12ter (painting)	-	-	-	-	-	-	BTA: Hamilton et al [48]
*HSFY2*	heat shock transcription factor Y-linked 2	transcriptional activators	Yp11-q12ter (painting)	-	-	-	-	-	-	BTA: Hamilton et al [48]
*KDM5D* ECA: *JARID1D*	lysine demethylase 5D	histone demethylase	-	-	-	-	-	Yq14	-	ECA: Raudsepp et al [60]
*MAP3K7/IP3Y*	mitogen-activated protein kinase 7 interacting protein 3 on Y	protein kinase	-	-	-	-	-	Yq14	-	ECA: Paria et al [62]
*MT-ND1Y*	miochondrially encoded NADH dehydrogenase1 on Y	catalytic activity	-	-	-	-	-	Yq14	-	ECA: Paria et al [62]
*NLGN4Y*	neuroligin 4 Y-linked	putative neuronal cell surface protein involved in cell-cell-interactions.	-	-	-	-	-	Yq14	-	ECA: Paria et al [62]
*OY1.1*	protein coding	-	Yp13 + Yp11-qter	-	-	-	-	-	-	BTA: Thomsen et al [41]
*OY11.1*	protein coding	-	Yp11-q12 ter	-	-	-	-	-	-	BTA: Thomsen et al [41]
*RBMY*	RNA binding motif protein Y-linked	binding protein	-	-	-	-	-	Yq14	-	ECA: Paria et al [62]
*RFX5Y*	regulatory factor X5 on Y	DNA-binding transcription activator activity	-	-	-	-	-	Yq14	-	ECA: Paria et al [62]
*RPS3AY*	ribosomal protein S3A	ribosomal protein	-	-	-	-	-	Yq14	-	ECA: Paria et al [62]
*SH2-A-1*	DNA linear STS	-	-	-	-	-	-	Yq14	-	ECA: Raudsepp et al [60]
*SH3-B-6*	DNA linear STS	-	-	-	-	-	-	Yq14	-	ECA: Raudsepp et al [60]
*SH3-B-7*	DNA linear STS	-	-	-	-	-	-	Yq14	-	ECA: Raudsepp et al [60]
*SRY*	sex determining region Y	transcriptional regulator	Yq12.3	Yq17	Yq12	Yq12	Yp12-p13	Yq14	-	BTA/BBU/OAR/CHI: Di Meo et al [45] SSC: Yang et al [100] ECA: Raudsepp et al [60]
*STSP1* ECA: *STS-Y*	steroid sulfatase (microsomal) pseudogene 1	protein kinase, sulfuric ester hydrolase activity	-	-	-	-	-	Yq14	-	ECA: Paria et al [62]
*TBL1Y*	transducin beta like 1Y-linked	transcription activation mediated by nuclear receptors	-	-	-	-	-	Yq14	-	ECA: Paria et al [62]
*TMSB4Y*	thymosin beta 4 Y-linked	actin monomer binding	-	-	-	-	-	Yq14	-	ECA: Paria et al [62]
*TSPY1* BTA-OAR-CHI-SSC-ECA: *TSPY*	testis specific protein Y-linked 1	nucleosome assembly protein	Yp13-p11 (painting)	-	Yp1.3-p1.2 (painting)	Yp1.3-p1.2 (painting)	Yp11-p12	Yq14	-	BTA/OAR/CHI: Hamilton et al [47]; Vogel et al [42]; Quilter et al [54] ECA: Raudsepp et al [60]
*TXLNGY* ECA: *CYorf15*	taxilin gamma pseudogene, Y-linked	syntaxin binding	-	-	-	-	-	Yq14	-	ECA: Paria et al [62]
*UBA1* SSC-ECA: *UBE1Y*	ubiquitin-like modifier-activating enzyme 1	ubiquitin activating enzyme activity	-	-	-	-	Yp11-p12	Yq14	-	SSC: Quilter et al [54] ECA: Paria et al [62]
*UMN0301*	microsatellite	-	Yp12.1-q11	-	-	-	-	-	-	BTA: Di Meo et al [45]
*UMN0304*	microsatellite	-	Yp12.1-q11	Yq11-q1.10	Yp11-q12	Yp11-q12	-	-	-	BTA/BBU/OAR/CHI: Di Meo et al [45]
*UMN0504*	microsatellite	-	Yp13-p12.2	Yq21-q22	-	-	-	-	-	BTA/BBU: Di Meo et al [45]
*USP9Y*	ubiquitin specific peptidase 9 Y-linked	ubiquitin-protein or polyubiquitin hydrolase	-	-	-	-	Yp12-p13	Yq14	-	SSC: Quilter et al [54] ECA: Raudsepp et al [60]
*UTY*	ubiquitously transcribed tetratricopeptide repeat containing, Y-linked	protein-protein interactions	-	-	-	-	Yp12-p13	Yq14-q15	-	SSC: Quilter et al [54] ECA: Raudsepp et al [60]
*Y2B17/YE1*	DNA linear STS	-	-	-	-	-	-	Yq14	-	ECA: Raudsepp et al [60]
*Y3B1*	DNA linear STS	-	-	-	-	-	-	Yq14	-	ECA: Raudsepp et al [60]
*Y3B12*	DNA linear STS	-	-	-	-	-	-	Yq14	-	ECA: Raudsepp et al [60]
*Y3B8*	DNA linear STS	-	-	-	-	-	-	Yq14	-	ECA: Raudsepp et al [60]
*YIR2*	inverted repeat 2Y	-	-	-	-	-	-	Yq14	-	ECA: Paria et al [62]
*YM2*	DNA linear STS	-	-	-	-	-	-	Yq14	-	ECA: Raudsepp et al [60]
*ZFY*	zinc finger protein Y-linked	DNA-binding transcription factor activity	Yp12.2	Yq110-q21	Yp1.2	Yp1.2	Yp12-p13	Yq14-q15	-	BTA/BBU/OAR/CHI: Di Meo et al [45] SSC: Quilter et al [54] ECA: Raudsepp et al [60]
*ZNF33bY*	zinc finger protein 33b on Y	regulation of transcription	-	-	-	-	-	Yq13 (painting)	-	ECA: Paria et al [62]
*λES6.0*	DNA segment	-	Yp12-p11	-	-	-	-	-	-	BTA: Goldammer et al [43]
TOTAL LOCUS = 89			BTA N = 18	BBU N = 5	OAR N = 5	CHI N = 5	SSC N = 10	ECA N = 46	VPA N = 0	

* The reported gene names follow the standards of the human gene nomenclature HGNC (Tweedie S, Braschi B, Gray KA, Jones TEM, Seal RL, Yates B, Bruford EA. Genenames.org: the HGNC and VGNC resources in 2021. Nucleic Acids Res. PMID: 33152070 PMCID: PMC7779007 DOI:10.1093/nar/gkaa980).

**Table 2:** Cytogenetic chromosome localisation of physically mapped PAR loci on the Y-chromosome of BTA, BBU, OAR, CHI, SSC, ECA and VPA, and their references

Locus			Physical localization							References
	
Symbol	Name^*^	Protein type	BTA	BBU	OAR	CHI	SSC	ECA	VPA	
*AKAP17A* ECA: *SFRS17A*	A-kinase anchoring protein 17A	A-kinase anchor protein 17A	-	-	-	-	-	Xp25tel/Yq15tel	-	ECA: Raudsepp and Chowdhary [63]
*ANOS1* BTA/OAR/CHI/ SSC/VPA: *KAL1*	anosmin 1	serine-type endopeptidase inhibitor activity	Xq43/Yp12.2-p13	-	Xp12/Yp12-p13	Xp12/Yp12-p13	Xp22.2/Yptel	-	Xp16/Yq11	BTA/OAR/CHI: Das et al [39] SSC: Das et al [55] VPA: Avila et al [74]
*ARSD*	arylsulfatase D	arylsulfatase D precursor	Xq43/Yp12.2-p13	-	Xp12/Yp12-p13	Xp12/Yp12-p13	-	Xp25tel/Yq15tel	-	BTA/OAR/CHI: Das et al [39] ECA: Raudsepp and Chowdhary [63]
ARSL BTA/OAR/CHI/ECA: *ARSE*	arylsulfatase L	arylsulfatase L precursor	Xq43/Yp12.2-p13	-	Xp12/Yp12-p13	Xp12/Yp12-p13	-	Xp25tel/Yq15tel	-	BTA/OAR/CHI: Das et al [39]; ECA: Raudsepp and Chowdhary [63]
*ARSF*	arylsulfatase F	arylsulfatase F precursor	Xq43/Yp12.2-p13	-	Xp12/Yp12-p13	Xp12/Yp12-p13	Xp24/Yp13	Xp25tel/Yq15tel	Xp16/Yq11	BTA/OAR/CHI: Das et al [39]; SSC: Das et al [55] ECA: Raudsepp and Chowdhary [63] VPA: Avila et al [74]
*ARSH*	arylsulfatase family member H	arylsulfatase H precursor	Xq43/Yp12.2-p13	-	Xp12/Yp12-p13	Xp12/Yp12-p13	-	Xp25tel/Yq15tel	-	BTA/OAR/CHI: Das et al [39]; ECA: Raudsepp and Chowdhary [63]
*ASMT*	acetylserotonin O-methyltransferase	methyltransferase activity	-	-	-	-	-	Xp25tel/Yq15tel	-	ECA: Raudsepp and Chowdhary, [63]
*ASMTL*	acetylserotonin O-methyltransferase like	O-methyltransferase activity	Xq43/Yp12.2-p13	Xq46/Yq21-q22	Xp12/Yp12-p13	Xp12/Yp12-p13	-	-	-	BTA/OAR/CHI: Das et al [39] BBU: Perucatti et al [52]
*CD99*	CD99 molecule (Xg blood group)	membrane glycoprotein	Xq43/Yp12.2-p13	-	Xp12/Yp12-p13	Xp12/Yp12-p13	-	Xp25tel/Yq15tel	-	BTA/OAR/CHI: Das et al [39] ECA: Raudsepp and Chowdhary [63]
*CLCN4*	chloride voltage-gated channel 4	membrane protein	-	-	-	-	-	-	Xp16/Yq11	VPA: Avila et al [74]
*CRLF2*	cytokine receptor like factor 2	membrane receptor	Xq43/Yp12.2-p13	-	Xp12/Yp12-p13	Xp12/Yp12-p13	Xp24/Yp13	Xp25tel/Yq15tel	-	BTA/OAR/CHI: Das et al [39] SSC: Das et al [55] ECA: Raudsepp and Chowdhary [63]
*CSF2RA*	colony stimulating factor 2 receptor subunit alpha	membrane receptor	Xq43/Yp12.2-p13	-	Xp12/Yp12-p13	-	Xp24/Yp13	-	Xp16/Yq11	BTA/OAR: Das et al [39]; Toder et al [34] SSC: Das et al [55]; VPA: Avila et al [74]
*DHRSX*	dehydrogenase/reductase X-linked	oxidoreductase activity	-	-	-	-	-	Xp25tel/Yq15tel	-	ECA: Raudsepp and Chowdhary [63]
*DU171056*	BES	-	Xq43.1/Yp12.2-p13	Xq46/Yq21-q22	Xp12/Yp12-p13	-	-	-	-	BTA/BBU/OAR: Perucatti et al [52]
*DXYS3 (TGLA325)*	DNA segment	-	Xq43.1q43.2/Yq12.2-q13	Xq46q47/Yq22	Xp12/Yp12-p13	Xp11-p14/Yp12-p13	-	-	-	BTA/BBU/OAR/CHI: Iannuzzi et al [51]; Di Meo et al [45] Piumi et al [50]
*DXYS4 (IOZARA1489)*	DNA segment	-	Xq43.1/Yp12.2	Xq46-q47	-	-	-	-	-	BTA/BBU: Prakash et al [49]
*EST BE750429*	DNA segment	-	Xq43.1/Yp12.2	Xq46–47/Yq21-q22	Xp12/Yp12	-	-	-	-	BTA/BBU/OAR: De Lorenzi et al [53]
*GPR143*	G protein-coupled receptor 143 pseudogene	G protein-coupled receptor activity	Xq43/Yp12.2-p13	-	Xp12/Yp12-p13	Xp12/Yp12-p13	Xp24/Yp13	-	Xp16/Yq11	BTA/OAR/CHI: Das et al [39]; SSC: Das et al [55] VPA: Avila et al [74]
*GTPBP6*	GTP binding protein 6 (putative)	metal ion binding	-	-	-	-	-	Xp25tel/Yq15tel	-	ECA: Raudsepp and Chowdhary [63]
*GYG2*	glycogenin 2	glycosyltransferase activity	Xq43/Yp12.2-p13	-	Xp12/Yp12-p13	Xp12/Yp12-p13	-	Xp25tel/Yq15tel	-	BTA/OAR/CHI: Das et al [39]; ECA: Raudsepp and Chowdhary [63]
*IL3RA*	interleukin 3 receptor subunit alpha	cytokine receptor activity	Xq43/Yp12.2-p13	-	Xp12/Yp12-p13	Xp12/Yp12-p13	-	-	-	BTA/OAR/CHI: Das et al [39]
*MID1*	midline 1	zinc ion binding	-	-	-	-	-	-	Xp16/Yq11	VPA: Avila et al [74]
*MXRA5*	matrix remodeling associated 5	protein peptidase inhibitor	Xq43/Yp12.2-p13	-	Xp12/Yp12-p13	Xp12/Yp12-p13	Xp24/Yp13	Xp25tel/Yq15tel	-	BTA/OAR/CHI: Das et al [39]; SSC: Das et al [55] ECA: Raudsepp and Chowdhary [63]
*NLGN4X* BTA/OAR/CHI: *NLGN4*	neuroligin 4 X-linked	hydrolases	Xq43/Yp12.2-p13	-	Xp12/Yp12-p13	Xp12/Yp12-p13	-	-	-	BTA/OAR/CHI: Das et al [39]
*OBP*	odorant binding protein (OBP porcine is pool of isoforms)	phosphoprotein binding	-	-	-	-	Xp24/Yp13	-	-	SSC: Skinner et al [56]
*PLCXD1*	phosphatidylinositol specific phospholipase C X domain containing 1	phosphoric diester hydrolase activity	-	-	-	-	-	Xp25tel/Yq15tel	-	ECA: Raudsepp and Chowdhary [63]
*PNPLA4*	patatin like phospholipase domain containing 4	triglyceride lipase activity	Xq43/Yp12.2-p13	-	Xp12/Yp12-p13	Xp12/Yp12-p13	Xp24/Yp13	-	Xp16/Yq11	BTA/OAR/CHI: Das et al [39] SSC: Chen et al [91]; Das et al [55] VPA: Avila et al [74]
*PPP2R3B*	protein phosphatase 2 regulatory subunit B″beta	protein kinase binding	Xq43/Yp12.2-p13	-	Xp12/Yp12-p13	Xp12/Yp12-p13	-	Xp25tel/Yq15tel	-	BTA/OAR/CHI: Das et al [39]; ECA: Raudsepp and Chowdhary [63]
*PRKXY*	protein kinase X-linked	cAMP-dependent protein kinase activity	Xq43/Yp12.2-p13	-	Xp12/Yp12-p13	Xp12/Yp12-p13	Xp24/Yp13	Xp25tel/Yq15tel	-	BTA/OAR/CHI: Das et al [39]; SSC: Das et al [55] ECA: Raudsepp and Chowdhary [63]
*PUDP* SSC: *HDHD1*	pseudouridine 5'-phosphatase	phosphatase protein	-	-	-	-	Xp24/Yp13	-	-	SSC: Das et al [55]
*SHOX*	short stature homeobox	DNA-binding transcription factor activity	-	-	-	-	Xp24/Yp13	-	-	SSC: Skinner et al [56]
*SHROOM2*	shroom family member 2	actin filament binding	-	-	-	-	Xp24/Yp13	-	Xp16/Yq11	SSC: Das et al [55] VPA: Avila et al [74]
*SLC25A6*	solute carrier family 25 member 6	carrier protein ADP/ATP	Xq42-q43.2/Yq12.2-q13	Xq46–47/Yq21–22	Xp12/Yp12-p13	Xp12/Yp12-p13	Xp24/Yp13	Xp25tel/Yq15tel	-	BTA/BBU/OAR/CHI: Das et al [39]; Di Meo et al [45] SSC: Das et al [55] ECA: Raudsepp and Chowdhary [63]
*STS*	steroid sulfatase	sulfuric ester hydrolase activity	Xq42-q43.2/Yq12.2-q13	-	Xp12/Yp12-p13	-	Xp24/Yp13	-	Xp16/Yq11	BTA: Das et al [39]; OAR: Toder et al [34] SSC: Das et al [55] VPA: Avila et al [74]
*SW949*	microsatellite	-	-	-	-	-	Xp24/Yp13	-	-	SSC: Das et al [55]
*TBL1X*	transducin beta like 1 X-linked	transcription corepressor activity	Xq43.1/Yp12.2	Xq46–47/Yq21-q22	Xp12/Yp12	-	Xp24/Yp13	-	-	BTA/BBU/OAR: De Lorenzi et al [53] SSC: Das et al [55]
*WWC3*	WWC family member 3	kinase binding	-	-	-	-	-	-	Xp16/Yq11	VPA: Avila et al [74]
*XG*	Xg glycoprotein (Xg blood group)	glycoprotein plasma membrane	-	-	-	-	-	Xp25tel/Yq15tel	-	ECA: Raudsepp and Chowdhary [63]
*ZBED1*	zinc finger BED-type containing 1	DNA-binding transcription protein	Xq43/Yp12.2-p13	-	Xp12/Yp12-p13	Xp12/Yp12-p13	-	Xp25tel/Yq15tel	-	BTA/OAR/CHI: Das et al [39]; ECA: Raudsepp and Chowdhary [63]
Total *LOCUS*			BTA N = 25	BBU N = 7	OAR N= 24	CHI N = 19	SSC N = 16	ECA N = 18	VPA N = 10	

* The reported gene names follow the standards of the human gene nomenclature HGNC (Tweedie S, Braschi B, Gray KA, Jones TEM, Seal RL, Yates B, Bruford EA. Genenames.org: the HGNC and VGNC resources in 2021. Nucleic Acids Res. PMID: 33152070 PMCID: PMC7779007 DOI:10.1093/nar/gkaa980).

## References

[1] Warr A, Affara N, Aken B (2020). An improved pig reference genome sequence to enable pig genetics and genomics research. GigaScience.

[2] Rosen BD, Bickhart DM, Schnabel RD (2020). De novo assembly of the cattle reference genome with single-molecule sequencing. GigaScience.

[3] Low WY, Tearle R, Bickhart DM (2019). Chromosome-level assembly of the water buffalo genome surpasses human and goat genomes in sequence contiguity. Nat Commun.

[4] Archibald AL, Cockett NE, Dalrymple BP (2010). The sheep genome reference sequence: a work in progress. Anim Genet.

[5] Bickhart DM, Rosen BD, Koren S (2017). Single-molecule sequencing and chromatin conformation capture enable de novo reference assembly of the domestic goat genome. Nat Genet.

[6] Chowdhary BP, Raudsepp T, Volff JN (2006). The horse genome. Vertebrate Genomes. Genome Dynamics.

[7] Fitak RR, Mohandesan E, Corander J, Burger PA (2016). The de novo genome assembly and annotation of a female domestic dromedary of North African origin. Mol Ecol Resour.

[8] Bianchi NO, Larramendy ML, Bianchi MS, Cortés L (1986). Karyological conservatism in South American camelids. Experientia.

[9] Di Berardino D, Nicodemo D, Coppola G (2006). Cytogenetic characterization of alpaca (Lama pacos, fam. Camelidae) prometaphase chromosomes. Cytogenet Genome Res.

[10] Frantz LAF, Bradley DG, Larson G, Orlando L (2020). Animal domestication in the era of ancient genomics. Nat Rev Genet.

[11] Fonseca PAS, Suárez-Vega A, Marras G, Cánovas Á (2020). GALLO: An R package for genomic annotation and integration of multiple data sources in livestock for positional candidate loci. Gigascience.

[12] Eusebi PG, Martinez A, Cortes O (2020). Genomic tools for effective conservation of livestock breed diversity. Diversity.

[13] Bai Y, Sartor M, Cavalcoli J (2012). Current status and future perspectives for sequencing livestock genomes. J Anim Sci Biotechnol.

[14] Sánchez-Molano E, Kapsona VV, Ilska JJ (2019). Genetic analysis of novel phenotypes for farm animal resilience to weather variability. BMC Genet.

[15] Liu R, Low WY, Tearle R (2019). New insights into mammalian sex chromosome structure and evolution using high-quality sequences from bovine X and Y chromosomes. BMC Genomics.

[16] Dhanoa JK, Mukhopadhyay CS, Arora JS (2016). Y-chromosomal genes affecting male fertility: a review. Vet World.

[17] Raudsepp T, Chowdhary BP (2015). The eutherian pseudoautosomal region. Cytogenet Genome Res.

[18] Skaletsky H, Kuroda-Kawaguchi T, Minx PJ (2003). The male-specific region of the human Y chromosome is a mosaic of discrete sequence classes. Nature.

[19] Rozen S, Skaletsky H, Marszalek JD (2003). Abundant gene conversion between arms of palindromes in human and ape Y chromosomes. Nature.

[20] Trombetta B, D’Atanasio E, Cruciani F (2017). Patterns of inter-chromosomal gene conversion on the male-specific region of the human Y Chromosome. Front Genet.

[21] Hughes JF, Skaletsky H, Brown LG (2012). Strict evolutionary conservation followed rapid gene loss on human and rhesus Y chromosomes. Nature.

[22] Soh YQ, Alföldi J, Pyntikova T (2014). Sequencing the mouse Y chromosome reveals convergent gene acquisition and amplification on both sex chromosomes. Cell.

[23] Skinner BM, Sargent CA, Churcher C (2016). The pig X and Y-chromosomes: structure, sequence, and evolution. Genome Res.

[24] Janečka JE, Davis BW, Ghosh S (2018). Horse Y chromosome assembly displays unique evolutionary features and putative stallion fertility genes. Nat Commun.

[25] De Lorenzi L, Parma P (2020). Identification of some errors in the genome assembly of bovidae by FISH. Cytogenet Genome Res.

[26] O'Connor RE, Fonseka G, Frodsham R (2017). Isolation of subtelomeric sequences of porcine chromosomes for translocation screening reveals errors in the pig genome assembly. Anim Genet 2017;48:395–403. Erratum in: Anim Genet.

[27] O'Connor C (2008). Fluorescence in situ hybridization (FISH). Nature Education.

[28] Bubendorf L, Jürgen Grote H, Syrjänen K, Bibbo M, Wilbur D (2008). CHAPTER 36 - Molecular techniques. Comprehensive cytopathology.

[29] Deakin JE, Potter S, O'Neill R (2019). Chromosomics: bridging the gap between genomes and chromosomes. Genes (Basel).

[30] O'Connor SJM, Turner KR, Barrans SL, Nielsen BS, Jones J (2020). Practical application of fluorescent in situ hybridization techniques in clinical diagnostic laboratories. In situ hybridization protocols Methods in molecular biology.

[31] Levsky JM, Singer RH (2003). Fluorescence in situ hybridization: past, present and future. J Cell Sci.

[32] Rubes J, Musilova P, Kopecna O, Kubickova S, Cernohorska H, Kulemsina AI (2012). Comparative molecular cytogenetics in cetartiodactyla. Cytogenet Genome Res.

[33] Graphodatsky AS, Trifonov VA, Stanyon R (2011). The genome diversity and karyotype evolution of mammals. Mol Cytogenet.

[34] Toder R, Gläser B, Schiebel K (1997). Genes located in and near the human pseudoautosomal region are located in the X-Y pairing region in dog and sheep. Chromosome Res.

[35] Yeh CC, Taylor JF, Gallagher DS, Sanders JO, Turner JW, Davis SK (1996). Genetic and physical mapping of the bovine X chromosome. Genomics.

[36] lannuzzi L (1994). Standard karyotype of the river buffalo (Bubalus bubalis L., 2n = 50). Report of the committee for the standardization of banded karyotypes of the river buffalo. Cytogenet Cell Genet.

[37] Cribiu EP, Di Berardino D, Di Meo GP (2001). International system for chromosome nomenclature of domestic bovids (ISCNDB 2000). Cytogenet Cell Genet.

[38] Gallagher DS, Womack JE (1992). Chromosome conservation in the bovidae. J Hered.

[39] Das PJ, Chowdhary BP, Raudsepp T (2009). Characterization of the bovine pseudoautosomal region and comparison with sheep, goat, and other mammalian pseudoautosomal regions. Cytogenet Genome Res.

[40] Perret J, Shia YC, Fries R, Vassart G, Georges M (1990). A polymorphic satellite sequence maps to the pericentric region of the bovine Y chromosome. Genomics.

[41] Thomsen PD, Jørgensen CB (1994). Distribution of two conserved, male-enriched repeat families on the Bos taurus Y chromosome. Mamm Genome.

[42] Vogel T, Borgmann S, Dechend F, Hecht W, Schmidtke J (1997). Conserved Y-chromosomal location of TSPY in Bovidae. Chromosome Res.

[43] Goldammer T, Brunner RM, Schwerin M (1997). Comparative analysis of Y chromosome structure in Bos taurus and B. indicus by FISH using region-specific, microdissected, and locus-specific DNA probes. Cytogenet Cell Genet.

[44] Weikard R, Kühn C, Brunner RM (2001). Sex determination in cattle based on simultaneous amplification of a new male-specific DNA sequence and an autosomal locus using the same primers. Mol Reprod Dev.

[45] Di Meo GP, Perucatti A, Floriot S (2005). Chromosome evolution and improved cytogenetic maps of the Y chromosome in cattle, zebu, river buffalo, sheep and goat. Chromosome Res.

[46] Habermann F, Winter A, Olsaker I, Reichert P, Fries R (2005). Validation of sperm sexing in the cattle (Bos taurus) by dual colour fluorescence in situ hybridization. J Anim Breed Genet.

[47] Hamilton CK, Favetta LA, Di Meo GP (2009). Copy number variation of testis-specific protein, Y-encoded (TSPY) in 14 different breeds of cattle (Bos taurus). Sex Dev.

[48] Hamilton CK, Revay T, Domander R, Favetta LA, King WA (2011). A large expansion of the HSFY gene family in cattle shows dispersion across Yq and testis-specific expression. PLoS One.

[49] Prakash B, Olsaker I, Gustavsson I, Chowdhary BP (1997). FISH mapping of three bovine cosmids to cattle, goat, sheep and buffalo X chromosomes. Hereditas.

[50] Piumi F, Schibler L, Vaiman D, Oustry A, Cribiu EP (1998). Comparative cytogenetic mapping reveals chromosome rearrangements between the X chromosomes of two closely related mammalian species (cattle and goats). Cytogenet Cell Genet.

[51] Iannuzzi L, Di Meo GP, Perucatti A, Incarnato D, Schibler L, Cribiu EP (2000). Comparative FISH mapping of bovid X chromosomes reveals homologies and divergences between the subfamilies bovinae and caprinae. Cytogenet Cell Genet.

[52] Perucatti A, Genualdo V, Iannuzzi A (2012). Advanced comparative cytogenetic analysis of X chromosomes in river buffalo, cattle, sheep, and human. Chromosome Res.

[53] De Lorenzi L, Genualdo V, Perucatti A, Iannuzzi A, Iannuzzi L, Parma P (2013). Physical mapping of 20 unmapped fragments of the btau_4.0 genome assembly in cattle, sheep and river buffalo. Cytogenet Genome Res.

[54] Quilter CR, Blott SC, Mileham AJ, Affara NA, Sargent CA, Griffin DK (2002). A mapping and evolutionary study of porcine sex chromosome genes. Mamm Genome.

[55] Das PJ, Mishra DK, Ghosh S (2013). Comparative organization and gene expression profiles of the porcine pseudoautosomal region. Cytogenet Genome Res.

[56] Skinner BM, Lachani K, Sargent CA, Affara NA (2013). Regions of XY homology in the pig X chromosome and the boundary of the pseudoautosomal region. BMC Genet.

[57] Cornefert-Jensen F, Hare WC, Abt DA (1968). Identification of the sex chromosomes of the domestic pig. J Hered.

[58] Grunwald D, Geffrotin C, Chardon P, Frelat G, Vaiman M (1986). Swine chromosomes: flow sorting and spot blot hybridization. Cytometry.

[59] Chowdhary BP, Paria N, Raudsepp T (2008). Potential applications of equine genomics in dissecting diseases and fertility. Anim Reprod Sci.

[60] Raudsepp T, Santani A, Wallner B (2004). A detailed physical map of the horse Y chromosome. Proc Natl Acad Sci USA.

[61] Paria N (2009). Discovery of candidate genes for stallion fertility from the horse Y chromosome [Doctoral Dissertation].

[62] Paria N, Raudsepp T, Pearks Wilkerson AJ (2011). A gene catalogue of the euchromatic male-specific region of the horse Y chromosome: comparison with human and other mammals. PLoS One.

[63] Raudsepp T, Chowdhary BP (2008). The horse pseudoautosomal region (PAR):characterization and comparison with the human, chimp and mouse PARs. Cytogenet Genome Res.

[64] Raudsepp T, Das PJ, Avila F, Chowdhary BP (2012). The pseudoautosomal region and sex chromosome aneuploidies in domestic species. Sex Dev.

[65] Raudsepp T, Gustafson-Seabury A, Durkin K (2008). A 4,103 marker integrated physical and comparative map of the horse genome. Cytogenet Genome Res.

[66] Chowdhary BP, Raudsepp T (2008). The horse genome derby: racing from map to whole genome sequence. Chromosome Res.

[67] Salvá B, Zumalacárregui J, Figueira AC, Osório MT, Mateo J (2009). Nutrient composition and technological quality of meat from alpacas reared in Peru. Meat Sci.

[68] Popova T, Tejedab L, Peñarrieta JM, Smith MA, Bush RD, Hopkins DL (2021). Meat of South American camelids - Sensory quality and nutritional composition. Meat Sci.

[69] Pauciullo A, Shuiep ET, Ogah MD, Cosenza G, Di Stasio L, Erhardt G (2019). Casein gene cluster in camelids: comparative genome analysis and new findings on haplotype variability and physical mapping. Front Genet.

[70] Morante R, Goyache F, Burgos A, Cervantes I, Pérez-Cabal MA, Gutiérrez JP (2009). Genetic improvement for alpaca fibre production in the Peruvian Altiplano: the Pacomarca experience. Anim Genet Res Inf.

[71] Cruz A, Morante R, Gutiérrez JP, Torres R, Burgos A, Cervantes I (2019). Genetic parameters for medullated fiber and its relationship with other productive traits in alpacas. Animal.

[72] Mendoza MN, Raudsepp T, More MJ, Gutiérrez GA, Ponce de León FA (2020). Cytogenetic mapping of 35 new markers in the alpaca (Vicugna pacos). Genes (Basel).

[73] Richardson MF, Munyard K, Croft LJ (2019). Chromosome-Level alpaca reference genome VicPac3.1 Improves genomic insight into the biology of new world camelids. Front Genet.

[74] Avila F, Baily MP, Perelman P (2014). A comprehensive whole-genome integrated cytogenetic map for the alpaca (Lama pacos). Cytogenet Genome Res.

[75] Jevit MJ, Davis BW, Castaneda C (2021). An 8.22 Mb assembly and annotation of the alpaca (Vicugna pacos) Y chromosome. Genes (Basel).

[76] Avila F, Das PJ, Kutzler M (2014). Development and application of camelid molecular cytogenetic tools. J Hered.

[77] Yue XP, Chang TC, DeJarnette JM, Marshall CE, Lei CZ, Liu WS (2013). Copy number variation of PRAMEY across breeds and its association with male fertility in Holstein sires. J Dairy Sci.

[78] Yue XP, Dechow C, Chang TC (2014). Copy number variations of the extensively amplified Y-linked genes, HSFY and ZNF 280BY, in cattle and their association with male reproductive traits in Holstein bulls. BMC Genomics.

[79] Pacheco HA, Rezende FM, Peñagaricano F (2020). Gene mapping and genomic prediction of bull fertility using sex chromosome markers. J Dairy Sci.

[80] Vogel T, Dechend F, Manz E (1997). Organization and expression of bovine TSPY. Mamm Genome.

[81] Wang M, Sun Z, Ding F (2021). Efficient TALEN-mediated gene knockin at the bovine Y chromosome and generation of a sex-reversal bovine. Cell Mol Life Sci.

[82] Xi J, Wang X, Zhang Y (2019). Sex control by Zfy siRNA in the dairy cattle. Anim Reprod Sci.

[83] Suriaty R, Mohd Hafiz AR, Halimaton Sa’adiah T, Mohd Hafizal A (2016). Detection of y chromosome of bovine Using testis specific protein and Amelogenin genes. Malaysian J Vet Res.

[84] Sánchez JM, Gómez-Redondo I, Browne JA, Planells B, Gutiérrez-Adán A, Lonergan P (2021). MicroRNAs in amniotic fluid and maternal blood plasma associated with sex determination and early gonad differentiation in cattle. Biol Reprod.

[85] Rhoads A, Au KF (2015). PacBio sequencing and its applications. Genomics Proteomics Bioinformatics.

[86] Pauciullo A, Fleck K, Lühken G, Di Berardino D, Erhardt G (2013). Dual-color high-resolution fiber-FISH analysis on lethal white syndrome carriers in sheep. Cytogenet Genome Res.

[87] Ye CJ, Heng HH, Wan T (2017). High resolution fiber-fluorescence in situ hybridization. Cancer cytogenetics Methods in molecular biology.

[88] Lindgren G (2001). Genome mapping in the horse [Dissertation].

[89] Wade CM, Giulotto E, Sigurdsson S (2009). Genome sequence, comparative analysis, and population genetics of the domestic horse. Science.

[90] Tomaszkiewicz M, Medvedev P, Makova KD (2017). Y and W Chromosome assemblies: approaches and discoveries. Trends Genet.

[91] Chen N, Bellott DW, Page DC, Clark AG (2012). Identification of avian W-linked contigs by short-read sequencing. BMC Genomics.

[92] Galtier N (2004). Recombination, GC-content and the human pseudoautosomal boundary paradox. Trends Genet.

[93] Katsura Y, Iwase M, Satta Y (2012). Evolution of genomic structures on mammalian sex chromosomes. Curr Genomics.

[94] Kutch IC, Fedorka KM (2018). Y-chromosomes can constrain adaptive evolution via epistatic interactions with other chromosomes. BMC Evol Biol.

[95] Bellott DW, Hughes JF, Skaletsky H (2014). Mammalian Y chromosomes retain widely expressed dosage-sensitive regulators. Nature.

[96] Jiang PP, Hartl DL, Lemos B (2010). Y not a dead end: epistatic interactions between Y-linked regulatory polymorphisms and genetic background affect global gene expression in Drosophila melanogaster. Genetics.

[97] Lemos B, Araripe LO, Hartl DL (2008). Polymorphic Y chromosomes harbor cryptic variation with manifold functional consequences. Science.

[98] Ohno S (1967). Sex chromosomes and sex-linked genes.

[99] Schenkel MA, Beukeboom LW, Pen I (2021). Epistatic interactions between sex chromosomes and autosomes can affect the stability of sex determination systems. J Evol Biol.

[100] Yang H, Fries R, Stranzinger G (1993). The sex-determining region Y (SRY) gene is mapped to p12-p13 of the Y chromosome in pig (Sus scrofa domestica) by in situ hybridization. Anim Genet.

